# Release of ice-nucleating particles from leaves during rainfall

**DOI:** 10.1007/s00114-025-01980-6

**Published:** 2025-03-25

**Authors:** Franz Conen, Annika Einbock

**Affiliations:** Department of Environmental Sciences, University of Basel, 4056 Basel, Switzerland

**Keywords:** Rainfall, Interception, Ice-nucleating particles, Wash off, Aerosolisation

## Abstract

Plant surfaces are a major source of particles able to initiate ice formation in clouds little below 0 °C. Rainfall promotes the release of such ice-nucleating particles (INPs) from vegetation. However, the pattern of their release throughout rain events remains unexplored. Here, we investigate at a high temporal resolution INP dynamics in rain impacting and running off single leaflets of *Juglans regia* during two rain events and relate the results to total INP inventories on the leaflets. Throughout both events, leaf runoff was distinctly enriched in INPs as compared with rainwater. A large fraction of INPs washed off during rainfall was released already at the beginning of the events. Except for the highest temperature interval, less than 40% of detachable INPs from leaf surfaces had been removed after the rain events. We hypothesise that surfactant excretion of INP-producing bacteria shapes the observed pattern of release.

**Supplementary Information:**

The online version contains supplementary material available at 10.1007/s00114-025-01980-6.

## Introduction

Vegetation intercepts and partitions rainwater. Most of it runs off plant surfaces, some evaporates and a tiny fraction is aerosolised through droplet splashing (Dunkerley 2009; Joung et al. 2017; Thoroddsen et al. 2012). Microorganisms dwelling on surfaces hit by raindrops are ejected with droplets (Gregory et al. 1959). These droplets can evaporate even during rainfall (Dunkerley 2009). If so, they leave previously immersed microorganisms behind, airborne. A tiny fraction of airborne particles, so-called ice-nucleating particles (INPs), facilitates the freezing of cloud droplets a little below 0 °C. Thereby, clouds are turned into an unstable mixture of vapour, liquid and ice, with effects on cloud development and precipitation (Hawker et al. 2021). A vast majority of atmospheric INPs active ≥  − 10 °C are heat sensitive, which indicates a predominantly biological origin (Testa et al. 2021; Conen et al. 2022). Leaf-derived INPs are an essential part of a postulated feedback between vegetation and rain: the bioprecipitation cycle (Morris et al. 2014). Earlier studies provided evidence for enhanced atmospheric concentration of INPs active between 0 and − 15 °C during rainfall (Huffman et al. 2013; Conen et al. 2017; Mignani et al. 2024), and the similarity of INPs splashed from *Betula pendula* during rainfall in situ with those on in vivo samples was demonstrated (Seifried et al. 2020). Furthermore, freezing spectra of particles collected at cloud height resemble those of particles washed off leaves (Einbock and Conen 2024). Yet, the dynamics of INP release from leaves over the course of a rainfall event have not been investigated. Approaching this question experimentally from the atmospheric side is difficult because of the large air volumes necessary to detect the rare atmospheric INPs. Here, we approach it from the source-side and sample rainwater running off a single leaf. We expect to find quantifiable differences in INPs between runoff from leaf (sample) and rainwater (control) from which to gain insights about the release of INPs from leaves over the course of a rain event.

## Material and methods

**Fig. 1:**
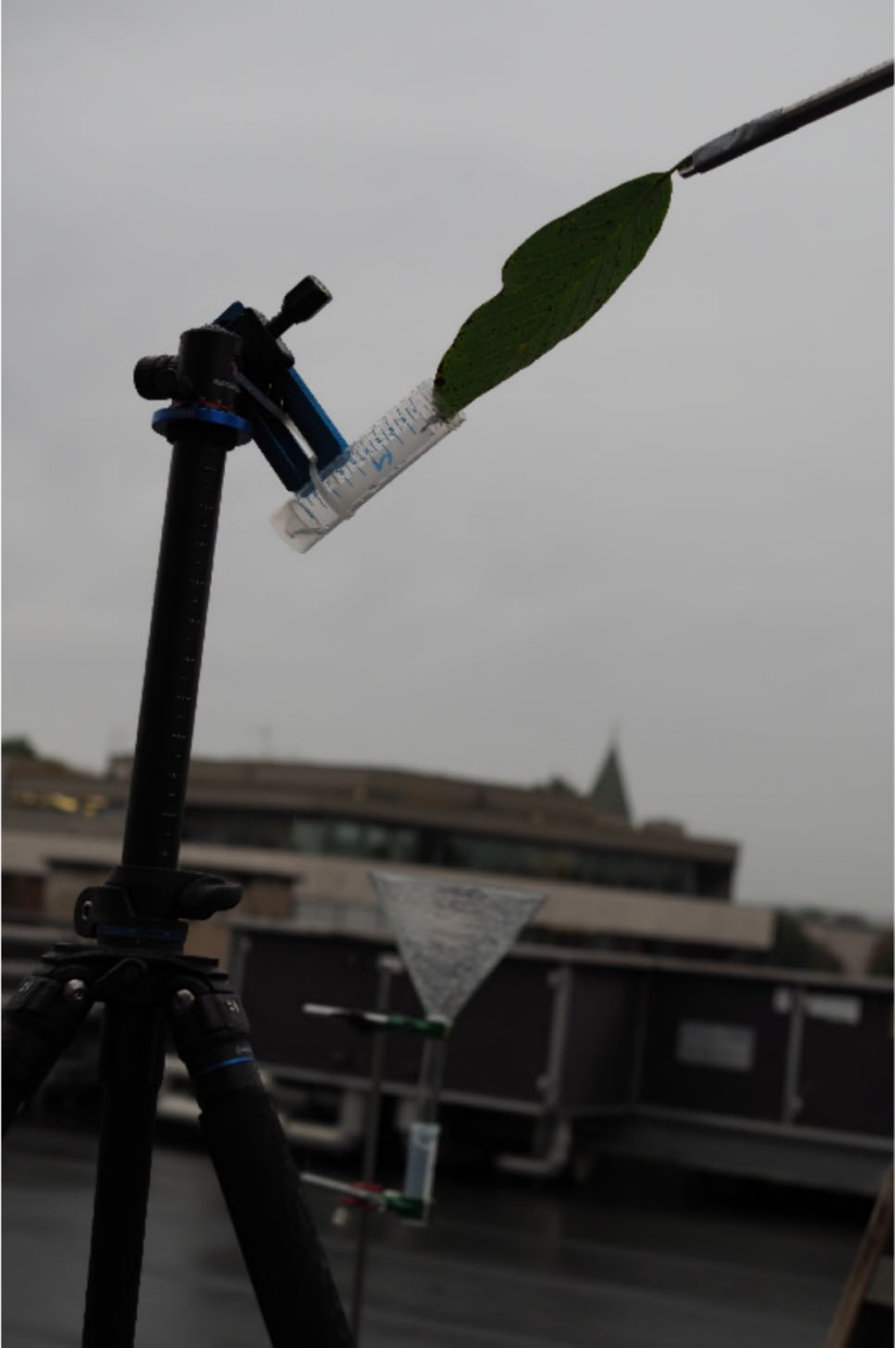
Experimental setup on the roof terrace of the Bernoullianum in Basel, Switzerland, on 8 October 2024. Rain was intercepted by a terminal leaflet of *Juglans regia* and running off into a 50-ml tube. When 2 to 3 ml had collected, the tube was replaced with an empty one. Parallel samples of rainwater were collected with a glass funnel

On two mornings in autumn 2024, we collected in a park 10 km northeast of Basel a green leaf from a walnut tree (*Juglans regia*) to expose its terminal leaflet a few hours later to the first hours of a natural rain event. Before the first event, no rain had been observed for at least 24 h, whereas the second leaf was collected in fog after a night with intermittent light rain. During the first event (*A*), on 11 September 2024, a total of 7.8 mm of rain fell between 12:45 and 18:39, and during the second event (*B*), on 8 October 2024, a total of 10.2 mm of rain fell between 09:33 and 13:23 local time. The leaflet area in events *A* and *B* was 114 and 124 cm^2^, respectively. Rainwater intercepted by the leaflet inclined about 30° from the horizontal run off into a 50-ml polypropylene tube. As soon as 2 to 3 ml had accumulated, the tube was replaced quickly with an empty one and put in a freezer (− 18 °C) for later analysis. For the same time intervals during which runoff was collected from the leaflet, we collected rainwater nearby with a glass funnel (20 cm diameter) as a control and for determining precipitation amounts (Fig. 1). Events *A* and *B* yielded a total of 22 and 24 parallel samples. After exposure to rain, INPs remaining on the leaflet were removed by immersing it in 45 ml of 0.1% NaCl in high-purity water (free of INPs), sonicating the tube for 1 min and shaking it by hand for another 20 s.

The concentration of INPs was analysed within 19 days (*A*) and 14 days (*B*) after the respective rain event. These analyses were done on a cold plate (CP-121HT Peltier-Thermoelectric Cold-Plate Cooler, TE Technology, Inc., MI, USA), with an uncertainty of ± 0.3 °C around the melting point of water. For each analysis, we covered the plate with a new piece of parafilm (10 cm × 13 cm) and pressed it for good contact onto the warm (45 °C) plate with a hand printing roller. Once cooled to below 25 °C, a total of 320 droplets, 5 µl each, were distributed onto the parafilm with an 8-channel electronic pipette. The plate was covered with a transparent acrylic lid, illuminated from a shallow angle with a LED line light and then cooled to − 5 °C. From visual observation, we can say that no freezing events had occurred above − 4 °C. After a minute, the first image of the droplet field was taken by a camera installed above the plate. The plate was cooled at a rate of 1.5 K min^−1^, and further images were taken after each 1 K step until − 11 °C. In these images, the number of frozen droplets, which are opaque and white as opposed to translucent liquid droplets, was counted, and the number concentration of INPs activated within each 1 K temperature interval was calculated according to Vali (2019). The absolute number of INPs released from the leaflet was calculated from the runoff volume and the difference in INP concentration in runoff from the leaflet and in rainwater collected with the glass funnel throughout the same time interval. In the following, we denominate INPs according to the centre of the temperature interval in which they were activated (e.g. INP_−4.5_ stands for INPs activated between − 4 and − 5 °C).

## Results

**Fig. 2:**
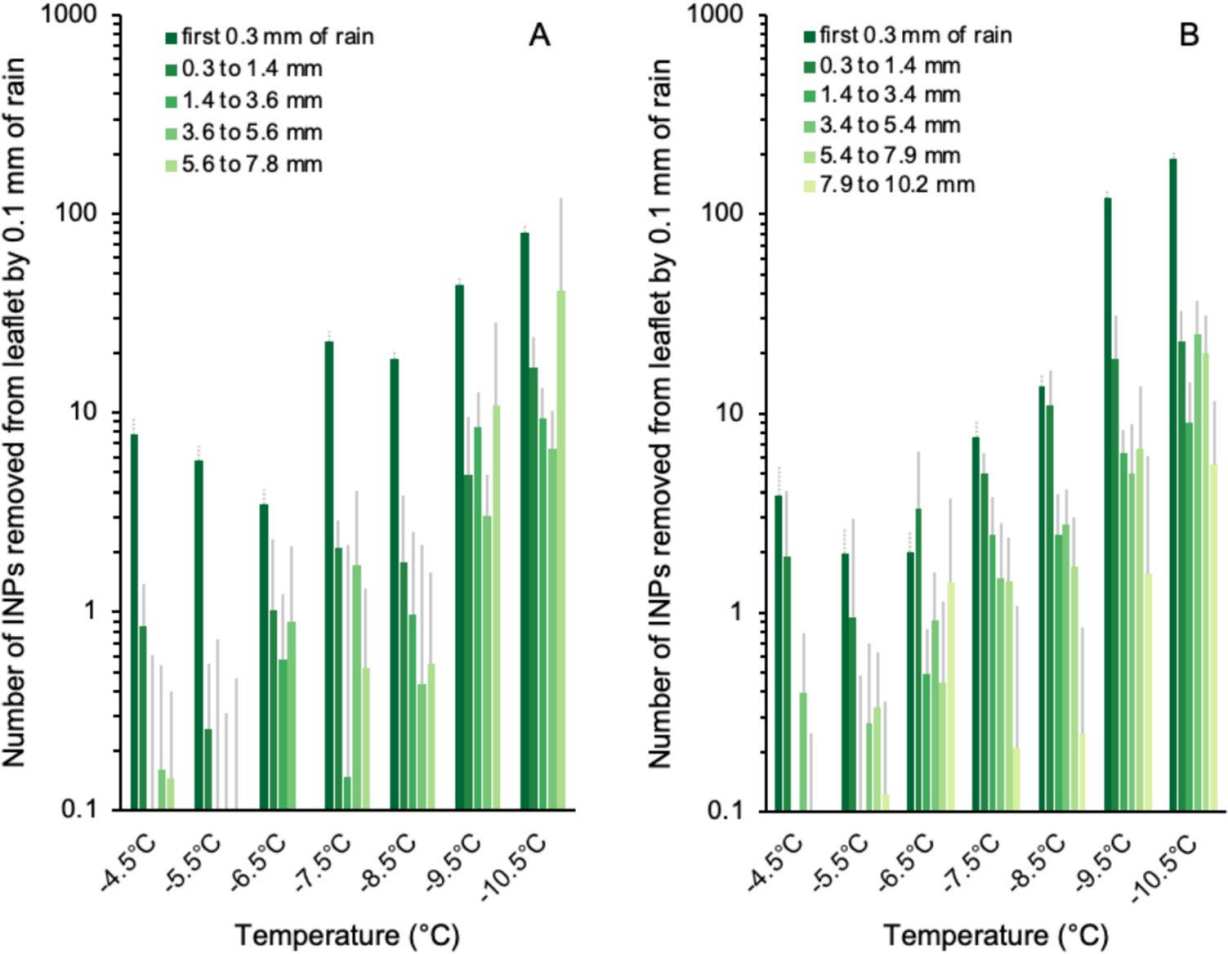
Differential ice nucleation spectra of particles released from *Juglans regia* leaflets during natural rain in events *A* (**A**) and *B* (**B**). Sampling resolution was on average 0.35 mm (standard deviation = 0.17 mm) during event *A* and 0.43 mm (standard deviation = 0.08 mm) during event *B*. Values for the first 0.3 mm of rain are based on a single sample, and error bars indicate the uncertainty associated with counting statistics in the number of frozen droplets. Consecutive intervals during the rain events are shown as the mean of 3 to 6 samples and error bars indicate one standard deviation of the mean

Rainwater became substantially enriched in INPs through contact with the leaflets. Water running off the leaflets had an average concentration of INP_−4.5_ that was 29 times greater than that of rainwater collected with the glass funnel. For INPs activated at colder temperatures, the difference was four- to sevenfold. Regarding only INPs released from the leaflets, we observed a decreasing trend throughout the course of both events (Fig. 2). In event *A*, runoff resulting from the first 0.3 mm of rain had by far the highest concentration of such INPs in all temperature bins. The relative decrease in INP concentration after the first 0.3 mm was less pronounced in event *B*, where the leaflet had already experienced light intermittent rain the night before. Overall, the differential freezing spectra of INPs released from the two leaflets were similar in that the concentration of INPs tended to increase with decreasing activation temperature. A small but notable exception was at the beginning of both events, where INP_−4.5_ had a higher concentration as compared with those in the next colder temperature interval (INP_−5.5_).

Sonicating the leaflets immediately after exposure to rainfall revealed the presence of INPs that had not been washed off by the total of 7.8 and 10.2 mm of rain in events *A* and *B*, respectively (Table 1). The number of these more sessile INPs was two to three times as large as the number of INPs washed off by rain, except for INP_−4.5_, of which about half had been washed off.

**Table 1:** Number of INPs released from *Juglans regia* leaflets (INPs per leaflet) by rain and by subsequent sonication

Dislocating force	Event	Freezing temperature (°C)						
		− 4.5	− 5.5	− 6.5	− 7.5	− 8.5	− 9.5	− 10.5
First 0.3 mm of rain	A	26	19	12	76	62	146	269
	B	12	6	6	24	43	380	591
All rain throughout entire event	A	41	20	53	147	123	690	1688
	B	40	35	116	198	319	1016	2162
Sonication in 45 ml of 0.1% NaCl in high purity water	A	28	57	172	354	463	1184	6059
	B	56	57	289	671	589	1874	6665

## Discussion

Similar numbers of INPs were removed from leaflets *A* and *B*. Earlier, we had eight times resampled two other walnut trees, sonicated one leaf from each tree and determined the total number density of INPs active ≥  − 10 °C (INP_≥−10_ cm^−2^) (Einbock and Conen 2024). There, successive samples from a particular tree were within a factor of 1.1 to 4.7, with a mean of 2.2. The two leaflets analysed here were, in terms of INP_≥−10_ cm^−2^, within a factor of 1.5. Yet, this similarity is of no importance, because we focus on relative changes with time and treatment. The observed release of INPs from *Juglans regia* leaflets during rain resembled the dynamics of particulate matter washed off a similar type of leaf. Xu et al. (2017) report exponentially decreasing wash-off rates with ongoing rainfall. In their treatment with the lowest rain intensity (15 mm h^−1^), 10 mm of rain had washed around 40% of the total particle mass from leaves of *Alianthus altissima*. Under much lower rain intensities in this study (*A*, 1.3 mm h^−1^; *B*, 2.7 mm h^−1^), an average of 32% (± 10%) of all INPs active within a temperature interval was removed from a leaflet by rain. By far, the largest fraction was released among the most efficient INPs (INP_−4.5_) during event *A* (59%). That INPs were washed off with similar ease as particulate matter that had been deposited from the atmosphere onto leaves indicates a weak attachment of INPs to the leaflet surface. Therefore, an association of the released INPs with microorganisms sheltered in biofilms seems implausible. Biofilms consist of aggregated microorganisms that are attached to a surface by an extracellular matrix (Thomas et al. 2024). A more obvious origin of the released INPs is microorganisms following another strategy than biofilm formation to avoid desiccation, namely the production and excretion of surfactants. These compounds adsorb to the outside of cells and to the leaf surface they are in contact with, but without attaching one to the other. Surfactants facilitate the formation of a thin water film when relative humidity is high (Burch et al. 2014). Cells on a water film are certainly more prone to be carried away with rainwater running over a leaf as compared with cells attached to the surface in a biofilm. *Pseudomonas syringae* is an epiphytic bacterium in which surfactant production and its effect on water availability was studied in detail (Hernandez and Lindow 2019). It is also a well-known producer of ice nucleation proteins (Lindow 2023). The freezing temperature of INPs produced by *P. syringae* can be as high as − 2 °C and decreases with the number of protein monomers in an INP aggregate (Qui et al. 2022). Likewise, the genus *Pantoea* includes species that produce INPs (Lindow 2023) and surfactants (Cloutier et al. 2021). Therefore, we suppose that INPs released from the leaflets during the two rain events were mostly associated with these kinds of organisms. This hypothesis is so far only supported by the observed wash-off behaviour. Further validation in future should be attempted by parallel surface tension measurements or comparative molecular genetic analyses of cells washed off by rain and removed by sonication.

Assuming the same concentration of INPs in leaf runoff as in droplets ejected from it by impacting raindrops, we can draw two conclusions regarding the aerosolisation of INPs during rainfall. First, leaves release a substantial fraction of the INPs they host already at the beginning of a rain event. Second, the number of INPs aerosolised with a certain amount of precipitation decreases throughout a rain event, but does not reach zero even after several hours of autumn rain. These initial findings are limited by replication. Further experiments in different seasons and with leaves also from other species are necessary to assess their generalisability.

## Supplementary Information

Below is the link to the electronic supplementary material.

ESM 1 (PDF 239 KB)

## Data Availability

Data on all samples collected and analysed in this study is available in the supplement.

## References

[CR1] Burch AY, Zeisler V, Yokota K, Schreiber L, Lindow SE (2014) The hygroscopic biosurfactant syringafactin produced by *Pseudomonas syringae* enhances fitness on leaf surfaces during fluctuating humidity. Environ Microbiol 16:2086–2098. 10.1111/1462-2920.12437 24571678 10.1111/1462-2920.12437

[CR2] Cloutier M, Prévost M-J, Lavoie S et al (2021) Total synthesis, isolation, surfactant properties, and biological evaluation of ananatosides and related macrodilactone-containing rhamnolipids. Chem Sci 12:7533. 10.1039/D1SC01146D 34163844 10.1039/d1sc01146d PMC8171317

[CR3] Conen F, Eckhardt S, Gundersen H, Stohl A, Yttri KE (2017) Rainfall drives atmospheric ice-nucleating particles in the coastal climate of Southern Norway. Atmos Chem Phys 17:11065–11073. 10.5194/acp-17-11065-2017

[CR4] Conen F, Einbock A, Mignani C, Hüglin C (2022) Measurement report: ice-nucleating particles active ≥ -15°C in free tropospheric air over Europe. Atmos Chem Phys 22:3433–3444. 10.5194/acp-22-3433-2022

[CR5] Dunkerley DL (2009) Evaporation of impact water droplets in interception processes: historical precedence of the hypothesis and a brief literature overview. J Hydrol 376:599–604. 10.1016/j.jhydrol.2009.08.004

[CR6] Einbock A, Conen F (2024) Similar freezing spectra of particles on plant canopies as in air at a high-altitude site. Biogeosciences 21:5219–5231. 10.5194/bg-21-5219-2024

[CR7] Gregory PH, Guthrie EJ, Bunce ME (1959) Experiments on splash dispersal of fungal spores. J Gen Microbiol 20:328–354. 10.1099/00221287-20-2-328 13654728 10.1099/00221287-20-2-328

[CR8] Hawker RE, Miltenberger AK, Wilkinson JM et al (2021) The temperature dependence of ice-nucleating particle concentrations affects the radiative properties of tropical convective cloud systems. Atmos Chem Phys 21:5439–5461. 10.5194/acp-21-5439-2021

[CR9] Hernandez MN, Lindow SE (2019) *Pseudomonas syringae* increases water availability in leaf microenvironments via production of hygroscopic syringafactin. Appl Environ Microbiol 85:e01014-e1019. 10.1128/AEM.01014-19 31285194 10.1128/AEM.01014-19 PMC6715840

[CR10] Huffman JA, Prenni AJ, DeMott PJ et al (2013) High concentrations of biological aerosol particles and ice nuclei during and after rain. Atmos Chem Phys 13:6151–6164. 10.5194/acp-13-6151-2013

[CR11] Joung YS, Ge Z, Buie CR (2017) Bioaerosol generation by raindrops on soil. Nat Commun 8:14668. 10.1038/ncomms14668 28267145 10.1038/ncomms14668 PMC5344306

[CR12] Lindow S (2023) History of discovery and environmental role of ice nucleating bacteria. Phytopathology 103:605–615. 10.1094/PHYTO-07-22-0256-IA 10.1094/PHYTO-07-22-0256-IA 36122194

[CR13] Mignani C, Hill TCJ, Nieto-Caballero M et al (2024) Ice-nucleating particles are emitted by raindrop impact. ESS Open Archive. 10.22541/essoar.172801408.80377732/v1

[CR14] Morris CE, Conen F, Huffman JA, Phillips V, Pöschl U, Sands DC (2014) Bioprecipitation: a feedback cycle linking earth history, ecosystem dynamics and land use through biological ice nucleators in the atmosphere. Glob Change Biol 20:341–351. 10.1111/gcb.12447 10.1111/gcb.12447 24399753

[CR15] Qiu Y, Hudait A, Molinero V (2022) How do size and aggregation of ice-binding proteins control their nucleation efficiency. J Am Chem Soc 141:7439–7452. 10.1021/jacs.9b01854 10.1021/jacs.9b01854 30977366

[CR16] Seifried TM, Bieber P, Felgitsch L et al (2020) Surfaces of silver birch (*Betula pendula*) are sources of biological ice nuclei: in vivo and in situ investigations. Biogeosciences 17:5655–5667. 10.5194/bg-17-5655-2020

[CR17] Testa B et al. (2021) Ice nucleating particle connections to regional Argentinian land surface emissions and weather during the Cloud, Aerosol, and Complex Terrain Interactions Experiment. J Geophys Res Atmos 126:e2021JD035186. 10.1029/2021JD035186

[CR18] Thomas G, Kay WT, Fones HN (2024) Life on a leaf: the epiphyte to pathogen continuum and interplay in the phyllosphere. BMC Biol 22:168. 10.1186/s12915-024-01967-1 39113027 10.1186/s12915-024-01967-1 PMC11304629

[CR19] Thoroddsen S, Takehara K, Etoh T (2012) Microsplashing by drop impacts. J Fluid Mech 706:560–570. 10.1017/jfm.2012.281

[CR20] Vali G (2019) Revisiting the differential freezing nucleus spectra derived from drop-freezing experiments: methods of calculation, applications, and confidence limits. Atmos Meas Tech 12:1219–1231. 10.5194/amt-12-1219-2019

[CR21] Xu X, Zhang Z, Bao L et al (2017) Influence of rainfall duration and intensity on particulate matter removal from plant leaves. Sci Total Environ 609:11–16. 10.1016/j.scitotenv.2017.07.141 28732292 10.1016/j.scitotenv.2017.07.141

