# Supplementary material for: Release of ice-nucleating particles from leaves during rainfall
Source: Naturwissenschaften. 2025 Mar 25;112(2):29. doi: 10.1007/s00114-025-01980-6 (PMC11937213; doi:10.1007/s00114-025-01980-6)
Supplement: Supplementary file 1 — (PDF 239 KB) [file 114_2025_1980_MOESM1_ESM.pdf]

The Science of Nature
Naturwissenschaften

Supplement to the article

Release of ice nucleating particles from leaves during rainfall

Franz Conen and Annika Einbock
Department of Environmental Sciences, University of Basel, 4056 Basel, Switzerland

Correspondence: Franz Conen (franz.conen@unibas.ch)

This supplement contains two Tables with the INP concentrations measured in rainwater and in runoff from the leaflets in events A (Tab. S1) and B (Tab. S2), and related parameters.

Tab. S1 Ice nucleating particles in rainwater and in leaf runoff collected during event *A* , and at the end of the experiment by sonicating the leaflet. The leaf was taken at 08:00 on 11.09.2024 from the lower, East-facing part of a mature tree behind Jazztone in Grütt Park, Lörrach. Leaf blade: 16.5 cm x 9.4 cm, 114 cm^2, dry mass 1.11 g

sample type	sampling interval		sample volume	cumulative rainfall	Concentration of INPs in sample (*k(T)* , see Vali 2019, Eq. 1) (INP mL^{-1} $°C^{-1}$)						
	from	to	(ml)	(mm)	-4.5°C	-5.5°C	-6.5°C	-7.5°C	-8.5°C	-9.5°C	-10.5°C
rain	12:45	14:10	10.4	0.3	0.0	0.0	0.6	0.0	0.0	0.0	10.3
leaf run-off	12:45	14:10	2.0		12.9	9.6	6.4	38.1	31.1	73.1	144.6
rain	14:10	14:24	9.6	0.6	0.0	0.0	0.0	0.0	0.0	0.0	1.9
leaf run-off	14:10	14:24	2.0		0.6	0.6	0.6	4.5	1.9	6.0	38.4
rain	14:24	14:55	7.5	0.9	0.0	0.0	0.0	0.0	0.0	0.0	0.6
leaf run-off	14:24	14:55	1.9		1.9	0.0	2.5	2.6	1.3	13.5	27.6
rain	14:55	15:18	8.2	1.1	0.0	0.0	0.6	4.4	2.6	3.3	1.3
leaf run-off	14:55	15:18	2.1		0.6	0.0	0.0	5.7	2.6	2.6	18.0
rain	15:18	15:40	8.4	1.4	0.0	0.0	0.6	0.0	0.0	0.0	0.6
leaf run-off	15:18	15:40	2.4		1.3	0.6	3.2	2.6	5.3	6.8	9.9
rain	15:40	16:00	7.0	1.6	0.0	0.0	0.6		0.0	0.0	0.6
leaf run-off	15:40	16:00	2.1		0.6	0.0	0.6	2.5	0.6	1.9	6.6
rain	16:00	16:27	34.5	2.7	0.6	1.3	0.0	2.5	0.6	1.3	3.3
leaf run-off	16:00	16:27	7.1		0.0	0.6	1.3	0.6	0.0	13.1	15.4
rain	16:27	16:40	8.3	3.0	0.0	0.0	0.6	0.0	0.0	1.3	4.5
leaf run-off	16:27	16:40	2.4		1.3	1.3	0.0	0.6	3.8	14.1	12.9
rain	16:40	17:02	9.8	3.3	0.0	1.3	0.0	1.3	0.6	0.0	3.8
leaf run-off	16:40	17:02	2.0		0.0	0.0	1.3	7.0	4.6	14.5	25.2
rain	17:02	17:12	10.7	3.6	0.0	0.0	0.6	0.6	0.6	1.3	2.6
leaf run-off	17:02	17:12	2.4		0.0	0.0	1.9	0.0	3.8	18.9	23.2
rain	17:12	17:17	10.0	4.0	0.0	0.0	1.3	0.6	1.3	3.8	14.8
leaf run-off	17:12	17:17	2.3		0.0	0.0	1.3	5.7	2.6	6.7	20.7
rain	17:17	17:27	9.4	4.3	0.0	0.0	0.0	1.3	2.5	0.6	2.6
leaf run-off	17:17	17:27	2.5		0.0	0.6	1.9	0.6	1.9	3.9	14.2
rain	17:27	17:36	11.0	4.6	0.0	0.6	0.6	0.6	1.3	3.2	5.9
leaf run-off	17:27	17:36	2.7		0.0	0.6	2.5	1.9	0.0	7.8	16.7
rain	17:36	17:45	9.8	4.9	0.0	0.0	0.6	0.6	4.5	1.3	5.9
leaf run-off	17:36	17:45	2.3		0.0	0.0	4.4	7.8	9.5	10.0	20.0
rain	17:45	17:54	10.1	5.2	0.0	0.0	0.6	0.6	1.3	2.5	7.8
leaf run-off	17:45	17:54	2.2		0.0	0.0	0.0	0.0	0.6	6.4	9.3
rain	17:54	18:00	10.6	5.6	0.0	0.6	0.6	0.0	1.3	1.3	1.3
leaf run-off	17:54	18:00	2.5		1.3	0.0	0.6	1.9	1.3	2.6	9.3
rain	18:00	18:08	11.9	6.0	0.0	0.6	0.0	0.0	4.4	2.6	2.0
leaf run-off	18:00	18:08	2.9		0.0	0.0	0.0	0.6	3.2	5.2	6.6
rain	18:08	18:13	11.3	6.3	0.0	0.0	0.6	0.0	1.3	0.6	3.2
leaf run-off	18:08	18:13	2.5		0.0	0.0	0.6	2.5	0.6	1.3	6.5
rain	18:13	18:21	12.4	6.7	0.0	0.0	0.0	1.3	0.0	1.9	4.5
leaf run-off	18:13	18:21	3.4		0.0	0.6	0.0	0.6	1.9	11.1	11.0
rain	18:21	18:27	9.0	7.0	0.0	0.6	0.6	1.3	0.6	0.0	4.5
leaf run-off	18:21	18:27	2.4		0.6	0.6	0.6	1.9	1.3	2.6	5.3
rain	18:27	18:33	11.6	7.4	0.0	0.0	0.0	1.3	0.0	2.5	3.2
leaf run-off	18:27	18:33	2.7		0.6	0.6	0.0	1.3	1.3	5.2	15.6
rain	18:33	18:39	13.0	7.8	0.0	0.0	0.0	0.6	0.6	2.5	2.6
leaf run-off	18:33	18:39	3.1		0.0	0.0	0.0	1.9	2.5	63.4	267.0
NaCl solution in which leaf was sonicated			45.0	-	0.6	1.3	3.8	7.9	10.3	26.3	134.7

Tab. S2 Ice nucleating particles in rainwater and in leaf runoff collected during event *B* , and at the end of the experiment by sonicating the leaflet. The leaf was taken at 06:30 on 08.10.2024 from the lower, North-facing part of the same tree as sampled for event *A* , Lörrach. Leaf blade: 18.3 cm x 8.7 cm, 124 cm^2, 1.04 g

sample type	sampling interval		sample volume	cumulative rainfall	Concentration of INPs in sample (*k(T)* , see Vali 2019, Eq. 1) (INP mL^{-1} $°C^{-1}$)						
	from	to	(ml)	(mm)	-4.5°C	-5.5°C	-6.5°C	-7.5°C	-8.5°C	-9.5°C	-10.5°C
rain	09:33	09:57	9.8	0.3	0.0	0.0	0.6	0.6	0.0	3.2	47.4
leaf run-off	09:33	09:57	3.2		3.8	1.9	2.6	8.0	13.3	122.0	232.1
rain	09:57	10:10	9.6	0.6	0.0	0.0	0.0	0.6	0.6	7.0	56.0
leaf run-off	09:57	10:10	2.4		1.3	4.5	9.2	9.0	15.4	20.8	82.4
rain	10:10	10:28	13.4	1.0	0.0	0.0	0.0	0.6	0.0	0.6	17.1
leaf run-off	10:10	10:28	2.6		0.6	0.0	3.2	7.1	9.5	20.4	41.6
rain	10:28	10:45	12.4	1.4	0.0	0.0	0.0	0.0	0.6	2.5	11.1
leaf run-off	10:28	10:45	2.7		6.3	0.0	2.6	7.3	24.5	50.3	60.5
rain	10:45	10:54	13.3	1.9	0.0	0.6	0.0	0.0	0.0	1.3	9.0
leaf run-off	10:45	10:54	3.1		0.0	0.0	0.6	6.4	2.6	11.5	27.5
rain	10:54	11:09	10.9	2.2	0.0	0.0	0.0	0.0	0.6	0.0	5.7
leaf run-off	10:54	11:09	2.7		0.0	0.6	0.6	1.9	4.5	10.0	15.6
rain	11:09	11:27	13.0	2.6	0.0	0.0	0.6	0.6	0.6	1.9	12.5
leaf run-off	11:09	11:27	3.1		0.0	0.6	1.9	2.5	6.5	6.7	13.5
rain	11:27	11:34	12.2	3.0	0.0	0.0	0.0	0.0	0.6	1.3	7.1
leaf run-off	11:27	11:34	3.0		0.0	0.0	0.6	2.5	1.3	7.8	18.8
rain	11:34	11:42	11.0	3.4	0.0	0.0	0.6	0.0	0.0	3.2	9.8
leaf run-off	11:34	11:42	2.8		0.0	0.0	0.6	3.2	3.2	13.4	27.4
rain	11:42	11:51	14.4	3.8	0.0	0.6	0.0	0.6	0.6	8.4	11.5
leaf run-off	11:42	11:51	3.4		0.6	0.6	1.3	1.9	3.2	14.8	40.1
rain	11:51	11:57	13.4	4.2	0.0	0.0	0.6	0.0	0.6	22.6	58.5
leaf run-off	11:51	11:57	3.1		1.3	1.3	3.2	3.2	6.0	21.3	68.0
rain	11:57	12:03	11.9	4.6	0.0	0.0	0.6	0.0	2.5	7.8	13.0
leaf run-off	11:57	12:03	3.1		0.0	0.6	0.6	0.0	5.1	15.4	53.2
rain	12:03	12:11	13.6	5.1	0.0	0.0	0.0	0.0	0.6	2.5	7.1
leaf run-off	12:03	12:11	3.7		0.6	0.0	1.3	3.8	5.9	13.7	50.6
rain	12:11	12:20	12.2	5.4	0.0	0.0	0.0	0.0	0.0	2.5	11.7
leaf run-off	12:11	12:20	3.7		0.0	0.0	0.6	0.6	1.3	8.4	41.7
rain	12:20	12:29	13.2	5.9	0.0	0.0	0.0	0.0	0.6	5.1	7.9
leaf run-off	12:20	12:29	3.6		0.0	0.6	1.9	2.5	0.6	3.9	52.1
rain	12:29	12:36	12.8	6.3	0.6	0.0	0.0	0.0	0.0	1.3	12.4
leaf run-off	12:29	12:36	3.6		0.0	0.0	0.0	0.0	2.5	9.1	22.4
rain	12:36	12:42	13.0	6.7	0.0	0.0	0.0	0.6	0.0	0.0	4.4
leaf run-off	12:36	12:42	3.3		0.0	0.6	0.0	2.5	4.5	22.7	28.9
rain	12:42	12:48	15.6	7.2	0.0	0.0	0.6	0.0	0.6	3.8	3.2
leaf run-off	12:42	12:48	4.7		0.0	0.0	0.6	2.5	2.6	7.9	18.2
rain	12:48	12:55	21.2	7.9	0.0	0.6	0.6	0.0	1.3	3.8	6.6
leaf run-off	12:48	12:55	6.1		0.0	1.3	1.3	1.3	2.6	10.6	29.2
rain	12:55	13:02	15.4	8.4	0.0	0.0	0.0	0.6	0.0	8.3	5.3
leaf run-off	12:55	13:02	4.0		0.0	0.0	0.6	0.0	0.6	2.5	9.8
rain	13:02	13:07	12.2	8.7	0.0	0.0	0.0	0.0	0.0	1.3	3.2
leaf run-off	13:02	13:07	3.3		0.0	0.0	0.0	0.0	0.0	5.7	12.6
rain	13:07	13:13	17.2	9.3	0.0	0.0	0.0	0.0	1.3	2.5	5.8
leaf run-off	13:07	13:13	4.6		0.0	0.6	1.3	0.6	1.3	3.9	7.9
rain	13:13	13:18	15.0	9.8	0.0	0.0	0.0	0.6	0.0	0.6	3.2
leaf run-off	13:13	13:18	4.2		0.0	0.0	0.0	0.0	1.3	1.9	3.8
rain	13:18	13:23	14.2	10.2	0.0	0.0	0.0	0.6	2.5	0.0	4.5
leaf run-off	13:18	13:23	3.6		0.0	0.0	7.0	2.6	2.0	9.5	23.6
NaCl solution in which leaf was sonicated			45.0	-	1.3	1.3	6.4	14.9	13.1	41.6	148.1
